# Studies on the Composition and Diversity of Seagrass *Ruppia sinensis* Rhizosphere Mmicroorganisms in the Yellow River Delta

**DOI:** 10.3390/plants12071435

**Published:** 2023-03-24

**Authors:** Shuai Shang, Liangyu Li, Hui Xiao, Jun Chen, Yu Zang, Jun Wang, Xuexi Tang

**Affiliations:** 1 School of Biological and Environmental Engineering, Binzhou University, Binzhou 256600, China; 2 College of Marine Life Sciences, Ocean University of China, Qingdao 266005, China; 3 First Institute of Oceanography, Department of Natural Resources, Qingdao 266061, China

**Keywords:** *Ruppia sinensis*, surrounding environment, seawater, rhizosphere microorganisms, Yellow River Delta

## Abstract

Seagrass is a significant primary producer of coastal ecosystems; however, the continued degradation of seagrass beds is a serious problem that has attracted widespread attention from researchers. Rhizosphere microorganisms affect seagrass and participate in many life activities of seagrass. This study explored the relationship between the composition of microbes in the rhizosphere and the surrounding environment of *Ruppia sinensis* by using High-throughput sequencing methods. The dominant bacterial groups in the rhizosphere surface sediments of *R. sinensis* and the surrounding environment are Proteobacteria, Bacteroidota, and Firmicutes. Moreover, the dominant fungal groups are Ascomycota, Basidiomycota, and Chytridiomycota. Significant differences (*p* < 0.05) were identified in microbial communities among different groups (rhizosphere, bulk sediment, and surrounding seawater). Seventy-four ASVs (For bacteria) and 48 ASVs (For fungal) were shared among seagrass rhizosphere, surrounding sediment, and seawater. The rhizosphere was enriched in sulfate-reducing bacteria and nitrogen-fixing bacteria. In general, we obtained the rhizosphere microbial community of *R. sinensis*, which provided extensive evidence of the relative contribution of the seagrass rhizosphere and the surrounding environment.

## 1. Introduction

Seagrass, a secondary entry from land to water, was adapted to the marine environment between 70 and 100 million years ago [1]. Compared to 250,000 species of terrestrial angiosperms, only 60 species of seagrass were adapted to the marine environment [2]. Seagrass meadows are one of the ecosystems with the richest biodiversity and highest productivity and ecological service function value in the earth’s biosphere. It provides habitats for many marine organisms and is essential for maintaining coastal ecosystems’ structural complexity, spatial heterogeneity, and functional stability [3]. Due to the interaction between land and sea, the seagrass meadows’ ecological environment is fragile and vulnerable to biological invasion. It faces worldwide degradation, which has attracted worldwide attention [4].

Complex and dynamic interactions between microorganisms and host plants can improve plant resistance to environmental stress [5]. Rhizosphere microorganisms play an important role in the growth and development of plants, promoting host plants’ growth, nutrient absorption, and stress resistance. Meanwhile, rhizosphere microorganisms can transport organic matter, nitrogen fixation, and denitrification [6]. The rhizosphere microbiome is also known as the plant’s second genome. The rhizosphere microbial assemblages are complicated and influenced by other biological and abiotic factors (soil pH value, soil salinity, soil conductivity, organic carbon, nitrogen, and phosphorus sources). Plants resist environmental changes by recruiting bacteria and fungi that are beneficial to them. These microorganisms influence the growth state of plants by regulating the secretion of plant hormones to improve nutrient absorption and produce volatile organic matter, thus improving the stress resistance of plants [7], and maintaining the dynamic stability of plants and rhizosphere microorganisms. The roots and rhizomes of seagrasses penetrate the sediment to form vast meadows. Seagrass roots can secrete amino acids, sugars, and oxygen to construct a complex relationship between seaweed and rhizosphere microbes [8]. Seagrass meadows form surface sediments and provide a specific environment for a rich and diverse microbial community [9]. Seagrass microorganisms play an essential role in maintaining the stability of the seagrass ecosystem [10]. There are many sulfide-metabolizing bacteria in seagrass, which can oxidize phytotoxic sulfides into non-toxic sulfates and play a vital role in the growth and development of seagrass [11]. The epiphytic microorganism can help seagrass resist pathogens and increase its ecological adaptability. Thus, microbial communities not only affect the growth and health of seagrass, but also control the succession of the seagrass community and even regulate the biochemical cycle of the seagrass bed, which plays an essential role in seagrass resistance to environmental changes [12]. As an essential supporting species of seagrass beds, *R. sinensis* is a widely distributed seagrass species in China and a vital support species of seagrass beds with crucial ecological functions. Due to the dual effects of global environmental change and human activities, *R. sinensis* faces population reductions due to anthropogenic impacts [13]. Meanwhile, *R. sinensis* is also widely distributed in the Yellow River Delta (YRD); the YRD is one of China’s three major estuarine deltas [14]. Furthermore, it is a typical coastal wetland ecosystem with high biodiversity. As an important component of biodiversity in the YRD, seagrasses play an essential role in maintaining the stability of the YRD ecosystem.

Several studies have been carried out on the microbial composition of *Zostera japonica* in the YRD [15], while the microbial communities of *R. sinensis* were still unknown. The present study explored the rhizospheric- and surrounding-environment microbial communities of *R. sinensis* using root, seawater, and sediment samples. Thus, we focused on characterizing each sample’s bacterial and fungal communities using high-throughput sequencing of 16S and ITS genes. We aimed to find the taxonomic composition characteristic of the *R. sinensis* microbiome.

## 2. Results

### 2.1. Characteristics of Environmental Factors in Seagrass Beds

In the present study, the One-way ANOVA method was used to analyze the environmental factors between the surrounding environment and the rhizosphere of seagrass beds. These results showed that the TOC and TS did not significantly differ between the BS and RS groups (*p* > 0.05). The EC was significantly different among the three groups and the order of the EC value was BS > RS > SW (*p* < 0.05). The pH in the SW group was significantly higher than that in the BS and RS groups (Table 1) (*p* < 0.05).

### 2.2. Alpha Diversity

**Figure 1:**
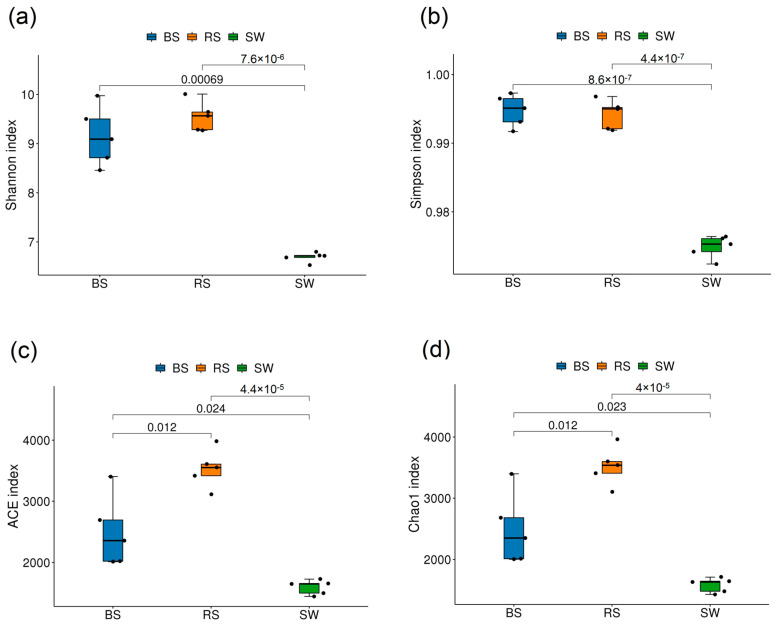
Alpha diversity of bacterial communities. RS: the seagrass rhizosphere soil samples; BS: sediment samples; SW: seawater samples. (**a**): Shannon index; (**b**): Simpson index; (**c**): ACE index; (**d**): Chao1 index. Different colored squares represent different groups. Statistical analysis was performed using the student’s *t*-test.

For bacteria, these results showed that the Shannon index and Simpson index of the SW group were significantly lower than that of the other groups (*p* < 0.05). According to the Ace index and Chao1 index, it can be seen that the order is RS > BS > SW, and there were significant differences among the three groups (*p* < 0.05) (Figure 1). 

**Figure 2:**
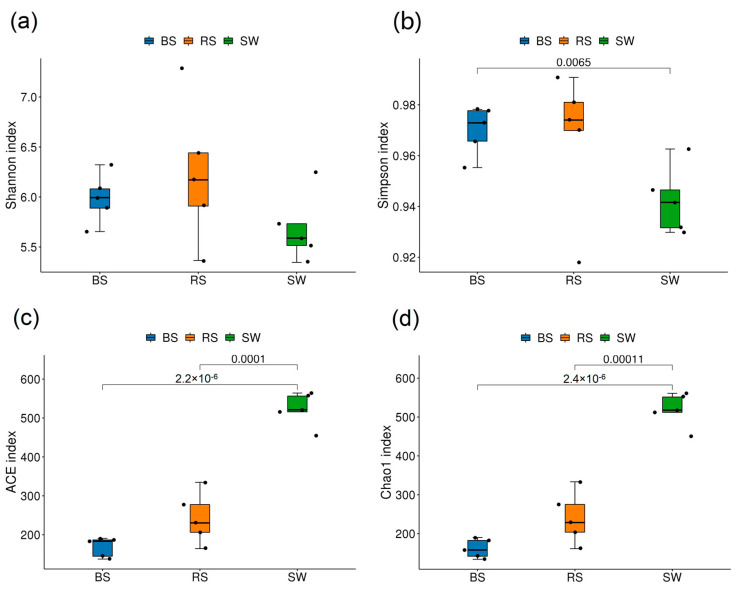
Alpha diversity of fungal community. RS: the seagrass rhizosphere soil samples; BS: sediment samples; SW: seawater samples. (**a**): Shannon index; (**b**): Simpson index; (**c**): ACE index; (**d**): Chao1 index. Different colored squares represent different groups. Statistical analysis was performed using the student’s *t*-test.

For bacteria, these results showed that the Shannon index and Simpson index of the SW group was significantly lower than that of the other groups (*p* > 0.05), while the Simpson index of the SW group was significantly lower than that of the BS group (*p* < 0.05). The Ace index and Chao1 index of the SW group are higher than those of the other two groups (Figure 2).

### 2.3. Beta Diversity

**Figure 3:**
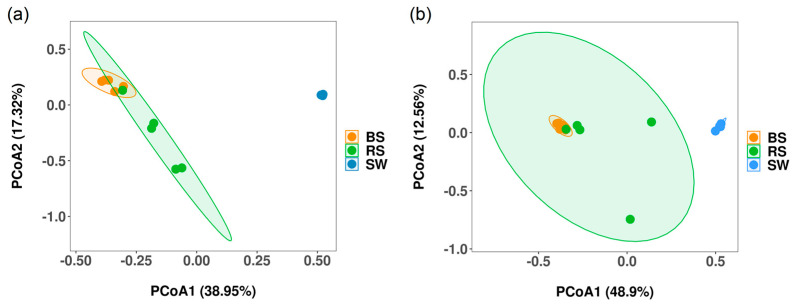
PCoA showing the bacterial (**a**) and fungal (**b**) community structure; RS: the seagrass rhizosphere soil samples; BS: sediment samples; SW: seawater samples. Different colored ellipsoids represent different groups.

We examined bacterial and fungal community structure differences between the seagrass rhizosphere and the surrounding environments. For bacteria, the PCoA results showed that the PC1 explained 38.95% of the variation, and the PC2 explained 17.32% of the variation, respectively. In total, 56.27% of the variation was explained by PC1 and PC2 (Figure 3a). For fungi, PC1 explained 48.90% of the variation, and PC2 explained 12.56% of the variation. In total, 61.46% of the variation was explained by PC1 and PC2 (Figure 3b). 

**Figure 4:**
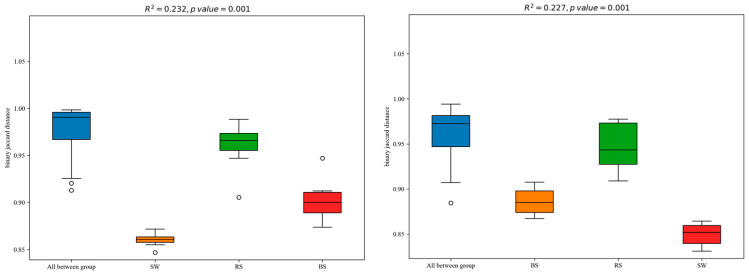
PERMANOVA analysis among different groups. The *R*-value is closer to 1, indicating that the difference between groups is more significant than the difference within groups; the smaller *R*-value indicates no significant difference between and within groups. RS: the seagrass rhizosphere soil samples; BS: sediment samples; SW: seawater samples.

The PERMANOVA was used to calculate the bacterial and fungal communities clustering among the three groups (Figure 4). These results showed that the different groups explained about 20% of the differences in bacterial and fungal community structure (R^2^ = 0.232, *p* = 0.001; R^2^ = 0.227, *p* = 0.001). 

### 2.4. Sequence Variants and Abundant Genera

**Figure 5:**
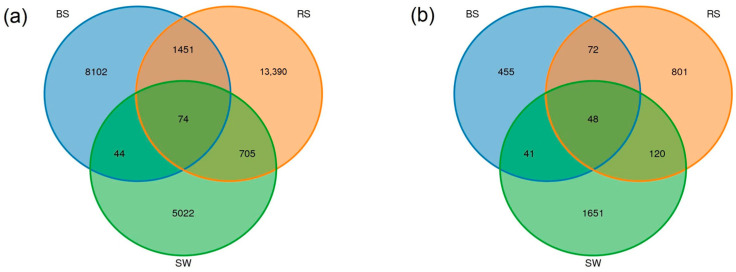
Venn diagrams showing the shared and unique bacterial (**a**) and fungal (**b**) ASVs among RS, BS and SW groups. RS: the seagrass rhizosphere soil samples; BS: sediment samples; SW: seawater samples. Each circle represents sampled compartments. Values within intersections represent shared ASVs, values outside intersections represent unique ASVs.

For bacteria, 74 ASVs were shared among seagrass rhizosphere, surrounding sediment, and seawater. The number of unique ASVs was 13,390, 8102, and 5022 in the RS, BS, and SW groups (Figure 5a). For fungi, the community shared 48 ASVs among the groups. The number of unique ASVs was 801, 455, and 1651 in the RS, BS, and SW groups (Figure 5b).

### 2.5. Composition of the Rhizosphere Microbial Community

**Figure 6:**
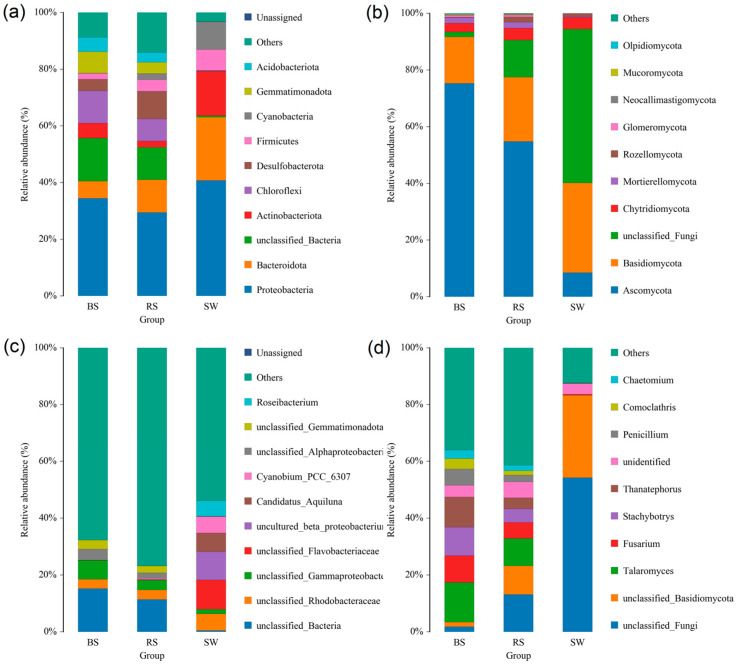
Relative abundance of bacterial (**a**,**c**) and fungal (**b**,**d**) communities at the phylum (**a**,**b**) and genus (**c**,**d**) level. RS: the seagrass rhizosphere soil samples; BS: sediment samples; SW: seawater samples.

The present study contained 1,935,998 raw reads and 2,063,349 raw reads in the bacteria and fungi, respectively. The generated serial number is PRJNA882191 and PRJNA881797. For bacteria, the dominant phyla were Proteobacteria, Bacteroidota, Actinobacteriota, Chloroflexi, Desulfobacterota, Firmicutes, Cyanobacteria, Gemmatimonadota, and Acidobacteriota in the RS group; the Proteobacteria, Bacteroidota, Actinobacteriota, Chloroflexi, Desulfobacterota, Firmicutes, Acidobacteriota, and Gemmatimonadota were dominant in the BS group; and the Proteobacteria, Bacteroidota, Actinobacteriota, Firmicutes, and Cyanobacteria were dominant in the SW group (Figure 6a). The Proteobacteria was the highest in the three groups. Except for the unclassified genera, *Roseibacterium*, *Candidatus_Aquiluna,* and *Cyanobium_PCC_6307* were dominant in three groups at the genus level (Figure 6c).

For fungi, the Ascomycota, Basidiomycota, Mortierellomycota, and Chytridiomycota were dominant in the RS group; the Ascomycota, Basidiomycota, Mortierellomycota, and Chytridiomycota were dominant in the BS group; and the Ascomycota, Basidiomycota, and Chytridiomycota were dominant in the SW group (Figure 6b). Except for the unclassified genera, *Talaromyces*, *Fusarium*, *Stachybotrys*, *Thanatephorus,* and *Penicillium* were dominant in the BS and RS groups at the genus level (Figure 6d). The *Ascomycota* was the highest in the BS and RS groups, while the *Basidiomycota* was the highest in the SW group.

## 3. Discussion

Plants and their microbiome together form a “holobiont” that interacts and coevolves with each other. Host plants provide a variety of microhabitats for microbial communities, and the microbes participate in various physiological processes of host plants. The surrounding environment is a vibrant microbial resource pool from host microorganisms [16]. 

Bacterial community diversity in the sediment and rhizosphere of seagrass was higher than in the surrounding seawater. The sediment and rhizosphere contain more nutrients than seawater and can protect microorganisms from sunlight and predation, leading to higher bacterial diversity in the rhizosphere and sediment [17]. Moreover, fungal community diversity in the sediment and rhizosphere of seagrass were similar. The similarity might be due to the proximity of the sediment and rhizosphere [18]. The Proteobacteria, Bacteroidota, Actinobacteriota, Chloroflexi, Desulfobacterota, Firmicutes, Cyanobacteria, Gemmatimonadota, and Acidobacteriota were dominant in the seagrass rhizosphere. Bacteroidota is known to be widely distributed across marine environments, and are decomposers of macromolecules, such as cellulose and chitin [19]. Nitrogen-fixing microorganisms are essential, including Proteobacteria, Firmicutes, and Actinobacteriota. Nitrogen fixation is essential in seagrass photosynthesis and balancing nitrogen loss. The relative stability of nitrogen-fixing flora in seagrass beds is crucial to seagrass beds’ health and ecological functions. 

A study of two tropical seagrasses *(Thalassia hemprichii* and *Enhalus acoroides*) found that the dominant bacterial communities involved sulfate cycling, nitrogen, and carbon fixing [20]. Several studies believed that Proteobacteria was the most abundant phylum [21] and was consistently enriched in the seagrass rhizosphere compared to bulk sediment [22]. A previous study found that the Proteobacteria were important in the low-molecular-weight substrates [23]. This study also found that the Proteobacteria was dominant in the seagrass rhizosphere, consistent with previous studies. In addition to providing fixed nitrogen to meet the needs of the rapid growth of seagrass, iron-reducing bacteria can also reduce sulfide toxicity by forming pyrite through long-term precipitation [24]. Previous studies found that sulfate-reducing bacteria are the main groups that degrade organic matter anaerobic in coastal oceans, and abundant organic carbon sources are conducive to their reproduction. Hence, they are more abundant in seagrass areas. In addition, the metabolic processes of sulfur and nitrogen are not independent of each other, and sulfate-reducing bacteria are also prominent members of nitrogen fixation in seagrass sediments [25]. Like other coastal marine ecosystems, microorganisms rapidly deplete oxygen in seagrass bed root sediments, resulting in an anoxic environment below the surface. In the present study, the sulfate-reducing bacteria occupy a crucial position in the seagrass rhizosphere ecosystem. Since a large amount of sulfate in the sediments can easily acquire electrons and become terminal electron acceptors, sulfuric acid Salt-reducing bacteria dominate the mineralization of organic matter. Geochemical evidence suggests sulfate reduction is closely related to carbon and nutrient cycling in seagrass bed sediments [26]. For fungi, Ascomycota was the most abundant in the BS and RS groups. It has been reported that fungi from Ascomycota represent the predominant microflora in marine environments. Penicillium, dominant in the rhizosphere and sediment, was demonstrated as common inhabitants of marine environments because these are adapted to peculiar chemical and physical conditions [27].

The study of microbial community organization is the basis for understanding the microbial ecosystem in seagrass beds, and its influencing factors are very complex. For bacteria, the Venn results showed that the 1524 common ASVs were identified between the RS and BS groups, and 779 common ASVs were identified between the SW and RS groups. Compared with the surrounding seawater, the microbiological compositions in the seagrass rhizosphere are more similar to the surrounding sediment. For fungi, 120 common ASVs were identified between the RS and BS groups, and 168 ASVs were identified between the SW and RS groups. Compared with the bacteria, the composition of fungi in the seagrass rhizosphere is more similar to that of the surrounding seawater. We speculated that the rhizosphere microbial community of *R. sinensis* was endemic and environment-acquired, not only from water bodies but also from sediments. A previous study found that compared with the perennial population, the annual *R. sinensis* population had a higher TOC [27]. Compared with the TOC of *R. sinensis* population, this research had a higher TOC.

Moreover, the fungal community structure of the seagrass rhizosphere is mainly related to TOC and PH in the surrounding seawater. The enrichment process of seagrass rhizosphere microbes could provide critical information on the interactions between seagrass and the surrounding environment [28]. A study of the *Z. marina* microbiome found that the bacterial leaf communities were similar to the seawater. The root bacterial communities were less heterogeneous and more distinct from the surrounding sediment [29].

## 4. Materials and Methods

### 4.1. Study Area

The study site was located in the YRD, Shandong, China. This area is located in the wetland at Bohai Sea, which widely distributes *Suaeda salsa*, *Tamarix chinensis* [30], Cynanchum chinense, *Spartina alterniflora*, and *R. sinensis*. The density of *R. sinensis*, it is the only seagrass species in the area. The seagrass rhizosphere, sediment, and seawater samples were collected from each site in the present study. The rhizosphere soil of *R. sinensis* was collected by first removing the associated plants, then excavating the whole solid goldenrod, followed by kneading the stalks of *R. sinensis*, then shaking the roots gently, and finally shaking off the soil. The rhizosphere soil of *R. sinensis* was obtained by following a previous study **[31]**. The seagrass rhizosphere soil samples were marked as the RS group; the sediment samples as the BS group; and the seawater samples as the SW group. All samples were divided into two parts; one part was used to determine soil and water physicochemical properties, and the other was used for DNA extraction.

### 4.2. Sediment and Seawater Physicochemical Properties Analysis

The present study determined the sediment and seagrass pH using a pH meter (Sartorius PB-10, Goettingen, Germany). Electrical conductivity (EC) was measured using a conductivity meter (Hanna HI98192, Villafranca Padovana, Italy). The Total Organic Carbon (TOC) and total sulfur (TS) were measured by following a previous study [31].

### 4.3. Molecular Methods

The DNA was extracted from the root, seawater, and sediment samples using the E.Z.N.A.**^®^** Soil DNA Kit (D4015, Omega, Inc., Syracuse, NY, USA). For bacteria, the primers of the V3–V4 region of the 16S ribosomal RNA gene was amplified by following a previous study [32]. For fungi, the primers of ITS1 were amplified using a previous study [33]. The Illumina Novaseq 6000 platform was used to obtain the raw data.

### 4.4. Statistical Analysis

In the present study, the raw data were denoised, and chimeric sequences were removed to get the final compelling data by using the Dada2 method in the QIIME2 2020.6 software [34]. The SILVA was used to classify for taxonomic annotation. The QIIME software generated the species abundance table for different taxonomic groups. The R was used to obtain the beta-diversity bar plots at each taxonomic level [35]. The Alpha diversity indices (AEC, Chao1, Shannon and Simpson indices) and beta diversity were obtained using the QIIME2 software [34]. The shared and unique amplicon sequence variants (ASVs) between the seagrass rhizosphere and the surrounding environment were shown by using the Venn diagrams. Principal coordinate analysis (PCoA) was used to visualize the difference in the microbial community structure between the seagrass rhizosphere, surrounding sediment, and seawater. The One-way ANOVA method and student’s *t*-test were used to calculate the differences in soil physicochemical properties and soil alpha diversity indices using SPSS 23.0 (IBM SPSS Inc., USA).

## 5. Conclusions

This study provides extensive evidence of the relative contribution of the seagrass rhizosphere and the surrounding environment. The dominant bacterial groups in the rhizosphere surface sediments of *R. sinensis* and the surrounding environment are Proteobacteria, Bacteroidota, and Firmicutes. Moreover, the dominant fungal groups are Ascomycota, Basidiomycota, and Chytridiomycota. Significant differences were identified in microbial communities among different groups (rhizosphere, bulk sediment, and surrounding seawater). The rhizosphere was enriched in sulfate-reducing bacteria and nitrogen-fixing bacteria.

## Figures and Tables

**Table 1:** Changes in the environmental factors among the BS, RS, and SW groups.

Groups	TOC (g/kg)	TS (g/kg)	EC	pH
BS	3.208 ± 0.297 a	0.44 ± 0.19 a	66.22 ± 6.40 a	7.81 ± 0.48 b
RS	3.096 ± 0.735 a	1.25 ± 0.40 a	37.66 ± 7.03 b	7.74 ± 0.16 b
SW	-	-	13.46 ± 0.40 c	8.60 ± 0.09 a

Values are the means ± standard error (*n* = 5). Lowercase letters in the same column indicate significant differences between the two groups (*p* < 0.05).

## Data Availability

The data that support the findings of this study are available in NCBI (accession number PRJNA882191 and PRJNA881797).
